# Phase Transformation Induced Self-Healing Behavior of Al-Ag Alloy

**DOI:** 10.3390/ma11020199

**Published:** 2018-01-27

**Authors:** Alena Michalcová, Ivo Marek, Anna Knaislová, Zdeněk Sofer, Dalibor Vojtěch

**Affiliations:** 1 Department of Metals and Corrosion Engineering, University of Chemistry and Technology in Prague, Technická 5, 166 28 Prague 6, Czech Republic; mareki@vscht.cz (I.M.); knaisloa@vscht.cz (A.K.); vojtechd@vscht.cz (D.V.); 2 Department of Inorganic Chemistry, University of Chemistry and Technology in Prague, Technická 5, 166 28 Prague 6, Czech Republic; soferz@vscht.cz

**Keywords:** self-healing, Al alloys, TEM

## Abstract

Self-healing alloys are promising materials that can decrease the consequences of accidents. To detect crack formation in a material is simple task that can be performed by e.g., sonic or ultrasound detection, but it is not always possible to immediately replace the damaged parts. In this situation, it is very advantageous to have the chance to heal the crack during operation, which can be done e.g., by annealing. In this paper, self-healing behavior was proven by TEM (Transmission electron microscope) observation of crack healing after annealing. The crack was observed in the rapidly solidified Al-30Ag alloy with non-equilibrium phase composition formed by a minor amount of Ag_2_Al and a supersaturated solid solution of Ag in an fcc-Al matrix (fcc = face centered cubic). After annealing at 450 °C, equilibrium phase composition was obtained by forming a higher amount of Ag_2_Al. This phase transformation did not allow the crack to be healed. Subsequent annealing at 550 °C caused recrystallization to a supersaturated solid solution of Ag in fcc-Al, followed by a return to the mixture of fcc-Al and Ag_2_Al by cooling, and this process was accompanied by the closing of the crack. This observation proved the self-healing possibilities of the Ag_2_Al phase. Practical application of this self-healing behavior could be achieved through the dispersion of fine Ag_2_Al particles in a structural material, which will enrich the material with self-healing properties.

## 1. Introduction

Self-healing behavior is a very promising property that can enhance the application ability of Al-based alloys. Al-based alloys have many advantages such as low density, good mechanical properties, and sufficient thermal resistivity if prepared by nonconventional methods like rapid solidification [1,2,3].

Self-healing properties, which means the ability of closing and healing crack initiated in a material during its utilization, have been described in cementous [4] and polymer materials [5]. The self-healing properties in Al-based alloys are mostly gained by surface modification [6,7,8,9] or by the creation of a composite material with some other smart material like NiTi [10,11]. Self-healing properties obtained by the encapsulation of a solder material into a metallic matrix have also been described [11,12,13]. Self-healing behavior was observed in a commercial Al alloy after suitable heat treatment [14] and some other precipitation-forming systems [15,16].

The aim of this article is to prove the feasibility of the self-healing process by phase transformation in Al alloy. Therefore, a rapidly solidified Al-30Ag alloy (composed of a supersaturated solid solution of Ag in an fcc-Al matrix (fcc = face centered cubic)) was chosen. This system provides two phase transformations [17]. The first one is the precipitation of the Ag_2_Al phase to equilibrium composition. The other is the high-temperature transformation to Ag solid solution in fcc-Al, which occurs after crossing the solvus line, at temperatures higher than 500 °C [17]. Ag_2_Al has an hcp structure (hcp = hexagonal close packet) [18], which means that during phase transformation there is no volume change. This is suitable, as this study aims to find a reversible self-healing mechanism, and volume changes could potentially speed up crack propagation. 

This research will hopefully lead to the development of novel alloys combining the advantages of both previously described self-healing Al alloys [11,14]. The self-healing mechanism obtained by the dispersion of Ag_2_Al nanoparticles in any Al structural alloy will be repeatable, unlike possible one-time self-healing by precipitation. Moreover, the composite of Al alloy and Ag_2_Al nanoparticles will be easy to prepare by powder metallurgy. The possible powder metallurgy processes for Al alloys are hot extrusion [3], spark plasma sintering [1], additive manufacturing [19,20], or cold pressure welding [21].

## 2. Materials and Methods

Rapidly solidified ribbons with the composition of Al-30 wt. % Ag (which corresponds to 10 at. % Ag) were prepared using a device designed by the Institute of Physics, Slovak Academy of Sciences (Bratislava, Skolakia), by the melt spinning technique with the circumferential velocity of the cooling wheel being 40 m/s. The specimen for TEM was prepared by ion polishing using PIPs (Precision Ion Polishing system) (Gatan, Pleasanton, CA, USA). The same specimen was annealed in a tube furnace (device designed by Department of Inorganic Chemistry, University of Chemistry and Technology in Prague, Prague, Czech Republic) in an Ar/H_2_ atmosphere at 450 °C for 1 h, and subsequently at 550 °C for 1h. The specimen was observed by a TEM Jeol 2200FS (Jeol, Akishima, Japan) equipped with an energy dispersive spectroscopy (= EDS) detector. The images were taken in scanning mode (= STEM) using a bright field (BF) and a dark field (HAADF) detector (Jeol, Akishima, Japan). The exact same section of the TEM specimen with the same crack was observed throughout this research.

The phase composition was studied by X-Ray diffraction using a PANalyticalX’Pert Pro device (Panalytical, Almelo, The Netherlands) with Cu–Kα radiation, with a step size of 0.017°2Theta and a measuring time per step of 40.64 s.

## 3. Results

**Figure 1:**
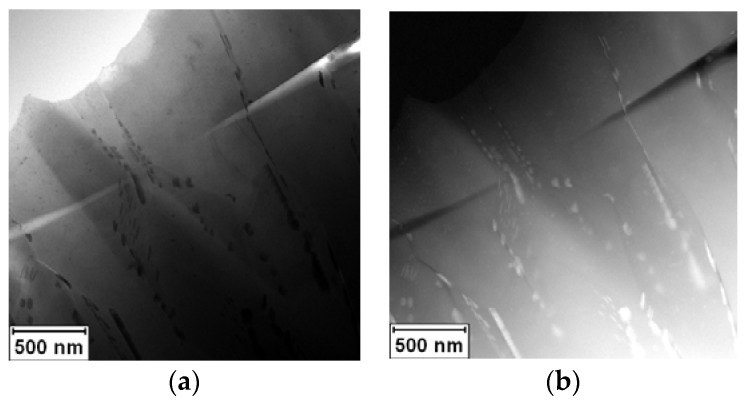
STEM micrograph of rapidly solidified Al-30Ag alloy: (**a**) BF; (**b**) HAADF.

**Figure 2:**
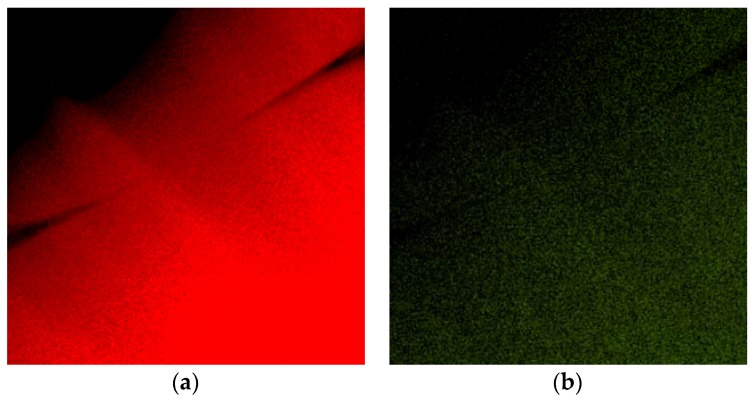
EDS elemental maps of area shown in Figure 1: (**a**) Al; (**b**) Ag.

The rapidly solidified Al-30Ag alloy exhibited non-equilibrium phase composition. It was formed by a minor Ag_2_Al phase and a supersaturated solid solution of Ag in an fcc-Al matrix, as is shown in Figure 1a,b. Figure 2a,b show the EDS (Energy Dispersive Spectrometer) elemental maps of Al and Ag, respectively. The presented phase composition is in agreement with previous observations by Dixmier et al. [21].

**Figure 3:**
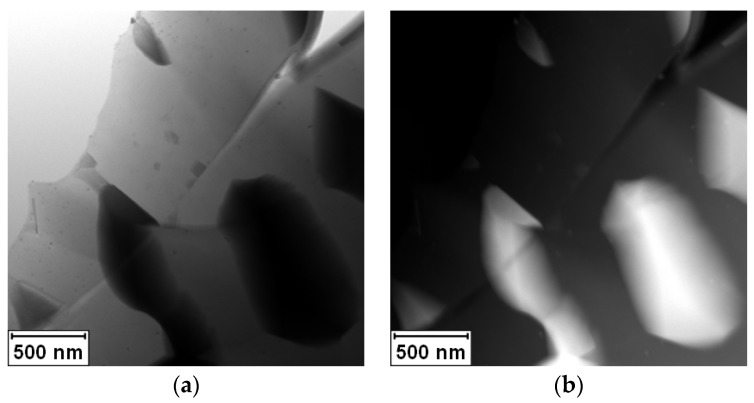
STEM micrograph of Al-30Ag alloy after annealing at 450 °C/1 h: (**a**) BF; (**b**) HAADF.

**Figure 4:**
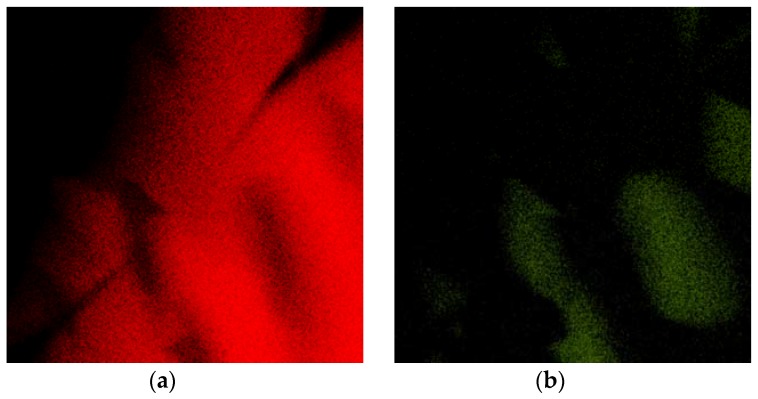
EDS elemental maps of area shown in Figure 3: (**a**) Al; (**b**) Ag.

The TEM samples were annealed at 450 °C for 1 h in an inert (Ar/H_2_) atmosphere, and exactly the same place was observed in order to see the microstructure evolution. The annealing caused an increase in the amount of Ag_2_Al phase and brought the alloy closer to equilibrium. Figure 3 documents that the crystallization of the Ag_2_Al phase did not lead to the closing of the crack. The crystallization of the Ag_2_Al phase is clearly visible in the elemental maps shown in Figure 4.

**Figure 5:**
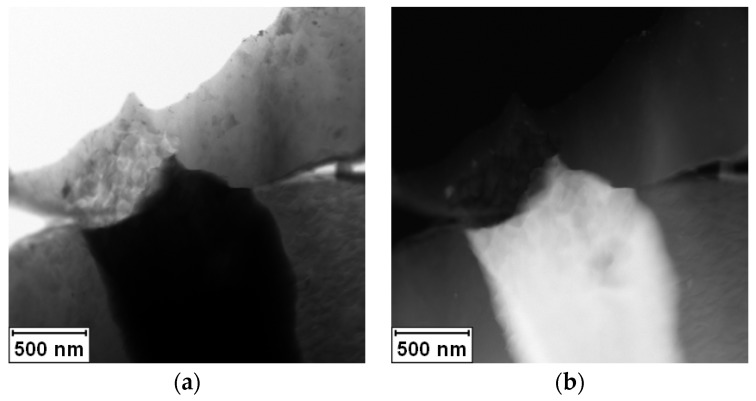
STEM micrograph of Al-30Ag alloy after annealing at 450 °C/1 h and 550 °C/1 h: (**a**) BF; (**b**) HAADF.

The same specimen was subsequently annealed at 550 °C for 1 h in an inert atmosphere. This temperature was chosen to cross the solvus line [17] to obtain a single-phase material at high temperatures. On cooling, the phase separation took place again. The phase transformation caused the closing of the crack inside the Ag_2_Al phase, as can be seen in Figure 5.

**Figure 6:**
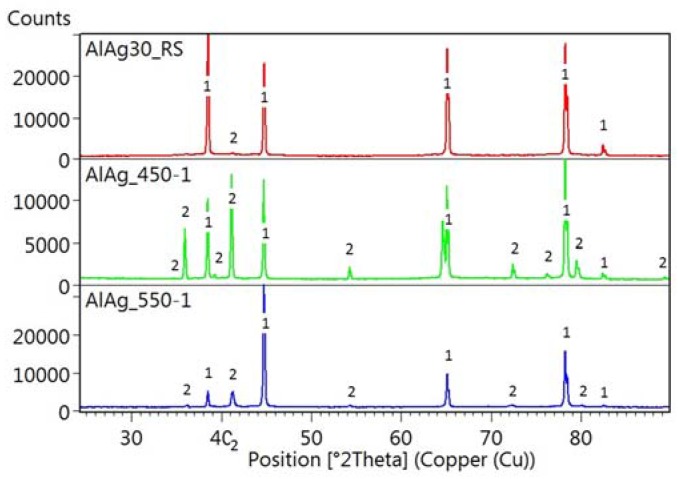
X-Ray diffraction pattern of Al-30Ag alloy after rapid solidification (red), after annealing at 450 °C/1 h and quenching (green), and after annealing at 550 °C/1 h and quenching (blue), 1 = fcc-Al, 2 = Ag_2_Al.

The changes of phase composition during heat treatment are shown in diffraction patterns in Figure 6. The highest pattern documents the phase composition of the rapidly solidified ribbon and proves that it is composed mainly of fcc-Al. The middle pattern gives the composition of the ribbon annealed at 450 °C for 1 h and quenched. It proves that a phase transformation to a mixture of fcc-Al and Ag_2_Al took place. The lowest pattern shows the phase composition of the ribbon annealed at 550 °C for 1 h and quenched. It documents the disappearance of Ag_2_Al at high temperatures as well as the recrystallization of the matrix by texture evolution (with the change of intensity of the peak belonging to fcc-Al).

## 4. Discussion

The Ag_2_Al phase exhibits self-healing properties when the solvus temperature is crossed (heating up to 550 °C for 1 h). This effect can be used for healing cracks in structural materials that cannot be immediately replaced after crack detection. Local annealing of cracks detected by sonic or ultrasound can decrease the risk of accidents. 

However, the Al-30Ag alloy provides no direct structural application due to its unsatisfactory mechanical properties. The results shown in this paper indicate the feasibility of doping structural Al-alloy with a fine dispersed Ag_2_Al phase, enriching the material with self-healing properties. This doping can be performed e.g., by the rapid solidification of Ag containing Al-alloy or by adding Ag nanoparticles to the Al-alloy.

Practical application of such self-healing properties can be achieved by the dispersion of fine Ag_2_Al particles in a structural material. The particles can be easily implemented to structural Al alloy during the powder metallurgy process e.g., by adding Ag nanoparticles. At sintering temperatures (400–500 °C) or by subsequent heat treatment, the Ag nanoparticles will react with the fcc-Al matrix, producing in situ a metal matrix composite. This process was also described for other systems such as Al-Si/CuAl_2_ [22]. It will ensure that the Ag_2_Al particles will be localized preferentially at the boundaries of former powder particles. Due to oxygen content, the boundaries tend to be the weakest part of a powder metallurgy-prepared material. Implementing Ag_2_Al particles with self-healing abilities at the relatively weak boundaries can significantly improve the properties of the material.

## 5. Conclusions

The aim of this paper was to describe possible self-healing behavior in an Al-Ag system. Because of this, Al with 30 wt. % of Ag was chosen. Rapidly solidified ribbons prepared from this alloy by the melt spinning technique were composed of a supersaturated solid solution of Ag in an fcc-Al matrix. The phase transformation occurring at low temperatures is the precipitation of Ag_2_Al from the supersaturated solid solution. The TEM observation revealed that this phase transformation did not lead to the healing of the crack presented in the TEM sample. After annealing at 450 °C for 1 h, the Ag_2_Al precipitated, but the crack in the sample was still present. Another phase transformation occurs at temperatures above 500 °C, namely, the transformation of Ag_2_Al into a saturated solid solution of Ag in an fcc-Al matrix. By observing the TEM sample annealed at 550 °C for 1 h, it was proven in this paper that the phase transformation of the Ag_2_Al phase into Ag solid solution in fcc-Al at 550 °C is able to close cracks inside the material, thus inducing the self-healing properties of the alloy. 

For the practical application of this phenomenon, the Ag_2_Al phase (nano) particles should be dispersed in a thermally stable Al-based structural alloy with good mechanical properties to form a composite material, the mechanical properties of which will be given by the composition of the Al alloy and the self-healing properties will be provided by the Ag_2_Al reinforcement.
